# Itsy Bitsy Spider…: Infants React with Increased Arousal to Spiders and Snakes

**DOI:** 10.3389/fpsyg.2017.01710

**Published:** 2017-10-18

**Authors:** Stefanie Hoehl, Kahl Hellmer, Maria Johansson, Gustaf Gredebäck

**Affiliations:** ^1^Max Planck Institute for Human Cognitive and Brain Sciences, Leipzig, Germany; ^2^Faculty of Psychology, University of Vienna, Vienna, Austria; ^3^Department of Psychology, Uppsala University, Uppsala, Sweden

**Keywords:** infants, pupillary dilation, arousal, fear, evolution

## Abstract

Attention biases have been reported for ancestral threats like spiders and snakes in infants, children, and adults. However, it is currently unclear whether these stimuli induce increased physiological arousal in infants. Here, 6-month-old infants were presented with pictures of spiders and flowers (Study 1, within-subjects), or snakes and fish (Study 1, within-subjects; Study 2, between-subjects). Infants’ pupillary responses linked to activation of the noradrenergic system were measured. Infants reacted with increased pupillary dilation indicating arousal to spiders and snakes compared with flowers and fish. Results support the notion of an evolved preparedness for developing fear of these ancestral threats.

## Introduction

Although clinical fears of spiders and snakes have a prevalence rate of 1–5 percent (Fredrikson et al., 1996), a strong dislike of these animals is reported by more than a third of the child population (Muris et al., 1997) and the adult population (Davey, 1991), the latter from which even entomologists are not exempt (Vetter, 2013). Fear of spiders and snakes are the most reported specific phobias, even though these animals hardly pose a threat to humans today (Russell, 1991; Fredrikson et al., 1996). Venomous spiders and snakes have, however, been dangerous for our ancestors for 40–60 million years of co-existence, possibly allowing primates to evolve mechanisms to quickly detect these potential threats (New and German, 2015).

Seligman (1971) proposed that primates possess an evolved preparedness to associate ancestral threats such as spiders and snakes with fear, thus explaining the high occurrence of specific phobias for these stimuli. Poulton and Menzies (2002) even suggested the existence of evolved fears of snakes and spiders that do not require fear-learning in ontogeny. These fears may, however, be extinguished through safe exposure and habituation in normal development explaining why specific phobias do not occur at an even higher prevalence rate. Evidence for arousal in response to snakes and spiders in early ontogeny would support the notion that an evolved mechanism underlies specific fears of ancestral threats in humans. In the present study, we therefore test whether young infants react with increased pupillary dilation to spiders and snakes.

Several studies have demonstrated rapid detection of spiders and snakes in visual search tasks with adult participants (Öhman et al., 2001), especially in patients with specific phobias (Pflugshaupt et al., 2005). Furthermore, adults are able to detect a single briefly presented task-irrelevant spider (but they less often detect houseflies or modern threats such as hypodermic needles) suggesting that the human visual system retains biases to reflectively direct attention toward this ancestral threat (New and German, 2015). There is also considerable evidence supporting the view that humans preferentially associate ancestral threats with fear. For instance, participants associate snakes and spiders more readily with aversive stimuli (e.g., mild electric shocks) than other stimuli, such as flowers, in fear conditioning experiments (see Öhman and Mineka, 2001, for a review). In addition, fear-associations with ancestral threats seem to be more robust and less prone to extinction than associations with non-threatening stimuli (Cook et al., 1986; Öhman and Mineka, 2001). However, these studies were conducted with adult participants, making it hard if not impossible to rule out the influence of prior socio-cultural learning experiences. In order to gain a better understanding of the origins of specific fears it is therefore important to test very young participants with limited prior learning opportunities.

Recent research has confirmed visual attention biases for stimuli representing an ancestral threat in infants as young as 5 months of age (Rakison and Derringer, 2008; LoBue and DeLoache, 2010). Similar to older children and adults (LoBue and DeLoache, 2008), infants are able to detect ancestral threats more quickly compared to non-threat-related images (LoBue and DeLoache, 2010). There is also some evidence that infants are prone to associate vocal and facial fear-expressions with spiders and snakes, but these effects were limited to girls in one study (Rakison, 2009) and dynamic stimuli showing snakes in motion in a second study (Deloache and LoBue, 2009). In another study, 9-month-old infants increased their attention to spiders (but not to flowers) when they were paired with fearful facial expressions, but showed an increased attention to snakes compared to fish regardless of the emotional context (Hoehl and Pauen, 2017). Thus, there are currently some hints, but no conclusive evidence for an evolved preparedness for building fear-associations with spiders and snakes in early human development.

The above-mentioned studies used measures of attention allocation (e.g., looking times) to test for early attention biases in human infants. In addition, physiological measures of arousal may be useful, as arousal is intricately linked with fear-conditioning and episodic memory consolidation (see Phelps and LeDoux, 2005, for a review). For instance, explicit memory of images rated as emotional (but not neutral) is enhanced through arousal induced by pain stimulation immediately after presentation of the images (Cahill et al., 2003). Thus, memory consolidation seems to be modified by arousal especially for stimuli that are predisposed to induce an emotional reaction. If evolutionary threats lead to increased arousal from early on in development, this might substantially support learning fear of these stimuli.

To our knowledge, only two studies on early biases for ancestral threats used arousal measures in infants. In the first study Erlich et al. (2013) found that 9-month-olds react with enhanced heart rate deceleration (indicating attentional orienting), larger startle eye-blinks and more visual orienting when listening to evolutionary fear-relevant sounds including angry voices, fire, and snake hissing, compared to modern fear-relevant sounds and pleasant sounds. However, this study did not control for all low-level properties of these sounds, making it difficult to separate a fear response from attention to, or processing of, a large array of other stimulus properties (i.e., to some extent results may reflect the perceived salience of stimuli based on acoustic cues such as dissonance, pitch, spectral tilt, and disharmonic fluctuations of pitch and loudness, rather than the perceived valence of equally salient stimuli).

Thrasher and LoBue (2016) measured 6- to 9-month-olds’ heart rate and startle eye blink response to videos of moving snakes or elephants paired with a happy or fearful voice. Infants’ heart rate was lower for snakes paired with a fearful voice compared to a happy voice while no such difference was found for elephants. Unexpectedly, startle magnitude was lower for snakes than for elephants, especially when paired with a fearful voice, making the results difficult to interpret. Furthermore, snakes and elephants were not matched for visual properties such as luminance and color.

Together, these two studies provide inconclusive evidence. This is partly due to the fact that startle eye-blink seems to be of limited use when working with infants as Erlich et al. (2013) report great difficulties obtaining these data. More conclusive evidence is still needed from a study using stimuli that control for low-level perceptual features and employing a physiological measure that can be readily applied with infant populations.

One way to achieve this is to measure physiological arousal via pupillary dilations. Pupillary responses, other than those of adjusting to ambient light, indicate activity of the noradrenergic system and therefore an aspect of the stress response (Laeng et al., 2012). Only a few studies looking at infants’ pupillary responses to negative or threatening stimuli exist in the literature and these focused on emotional expressions. In one recent study, 14- to 17-month-olds showed increased pupil dilation to negative (fearful and sad) vs. positive or neutral facial expressions, indicating sensitivity to the valence of facial expressions in this age group (Aktar et al., 2016). Similarly, Gredebäck et al. (2012) report a trend for increased pupillary responses for fearful vs. neutral and happy faces in 14-month-olds. Six and twelve month old infants also reacted with increased pupillary dilation when seeing and hearing a peer in distress compared to a neutral condition (Geangu et al., 2011). In contrast to these findings, Jessen et al. (2016) found increased pupillary responses to happy vs. fearful facial expressions when using very short presentation times in 7-month-old infants. The authors attribute the discrepancy of their findings with previous research on differing presentation modes and stimulus durations. Finally, Hepach and Westermann (2013) report that 14- but not 10-month-old infants respond with increased pupillary dilation to actions that are incongruent with an actor’s expressed emotions, i.e., a tender action accompanied by an angry facial expression and an aggressive action accompanied by a happy expression, suggesting that between 10 and 14 months infants become sensitive to the congruence of other people’s actions and emotional expressions. In sum, previous research confirms that pupillary measures are a useful tool to investigate arousal in response to emotional stimuli in infants, with a specific sensitivity of infant pupillary dilation to negative stimuli.

Here, we use pupillary dilation to investigate whether 6-month-old infants react to visual displays of spiders and snakes with increased arousal compared to fear-irrelevant images matched for color, luminance, and size. Assuming an evolved preparedness to develop fear for ancestral threats (Seligman, 1971), we predict increased pupillary dilation for spiders and snakes when compared to visually matched control stimuli that do not represent an ancestral threat to humans (i.e., flowers and fish).

## Study 1

We conducted two studies with 6-month-old infants measuring pupillary dilation using a Tobii T120 near infrared eye tracker. In Study 1 infants saw pictures of spiders and flowers (spider-flower experiment) and pictures of snakes and fish (snake-fish experiment). The order of both experiments was counterbalanced across participants and stimuli from the two categories within each experiment were presented intermixed in a pseudorandom order. Pupillometric data from the spider stimulus category were compared with the neutral flower stimulus category, and snake stimulus category data were compared with fish stimulus category. All experiments were conducted with the understanding and written and oral informed consent of each participant’s parent. The local ethics committee declared this study exempt. The committee stated that it would not consider the application since it does not need ethical approval. All experiments were conducted with the understanding and informed consent of each participant’s parent in accordance with the Declaration of Helsinki.

### Materials and Methods

#### Participants

Sample sizes (*N* = 16 per study/experiment) were pre-determined based on *a priori* power analysis (G^∗^Power: Faul et al., 2007) given a level of significance of 0.05, power of 0.8 and expected (moderate) effect size of *f* = 0.25. Study 1 included 16 6-month-old infants (*M* age = 183 days, *SD* = 3.7, 11 boys). All participants were included in the final analysis. All participants were recruited by means of their parents responding to an invitation letter sent to families with children of appropriate age living in Uppsala, a medium-size Swedish city.

#### Material and Apparatus

**FIGURE 1:**
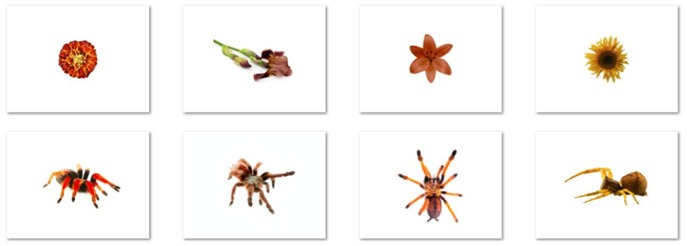
Four pairs from the total of eight color-matched pairs of flowers **(Top)** and spiders **(Bottom)** used in Study 1. Each item from the spider category has a corresponding color-matched flower item.

**FIGURE 2:**
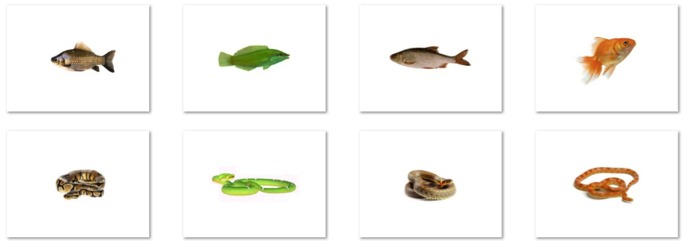
Four pairs from the total of eight color-matched pairs of fish **(Top)** and snakes **(Bottom)** used in Studies 1 and 2. Each item from the snake category has a corresponding color-matched fish item.

Two sets of items were used, one consisting of eight photographs each of spiders and flowers (16 total), and one consisting of eight photographs each of snakes and fish (16 total). Flowers and fish were chosen for comparison because they can be relatively easily matched in terms of low-level properties with spiders and snakes, respectively, due to similar morphology and surface properties. Each item in both sets had a corresponding color-matched item from the other category (**Figures 1**, **2**).

The color-matching was performed firstly by choosing appropriate pairings from the original coloring of the photographs, and secondly using photo editing software to apply the color content of one of the photographs onto the other. Thereby, the color content of one photograph was duplicated onto the other, rendering them pair-wise identical in regards to color. Across all pairs in both sets, the size of each item on screen was adjusted to be identical (60,000 pixels; ± 1,000 pixels). Lastly, the luminosity was leveled to ensure that brightness (and the confounding light-induced pupillary constriction) was even across each set (245 luminosity units). This was to ensure that the total amount of light in each item was identical.

Items from the two sets were presented once each in counter-balanced orders on a Tobii T120 near infrared eye tracker (sampling rate = 60 Hz, accuracy = 0.5 degrees, monitor size = 17 inches; Tobii, Stockholm, Sweden).

#### Procedure

Study 1 was conducted in two phases with a short break in between, to make sure that the infant was not fatigued. The content of each phase was counter-balanced, i.e., half of the participants received the spider-flower experiment in the first phase and the snake-fish experiment in the second phase, the other half received experiments in the reversed order. Within each set order, the first stimulus presented in the pseudo-randomized (pre-determined) order was equally often a snake or a spider as it was a fish or a flower. Per condition, 8 trials were run, each featuring a different exemplar of the respective category, presented individually. Each stimulus was presented for 5 s. Prior to each stimulus there was a 3 s long white screen.

All experiments were conducted in the same window-less room with constant ambient light across all participants. Infants were seated in their parent’s lap with a distance of 60 cm from the eye-tracker’s screen. Parents were required to wear opaque glasses (sunglasses covered with a non-transparent plastic sheet) to ensure that they did not see the stimuli. They were told beforehand what the pictures would contain and were shown example images after the experiment.

The experiment started directly after a short 5-point gaze calibration after which infants were already attending the screen. Upon the experiment’s start, trials were presented regardless of infants’ gaze direction and therefore a short attention grabber to keep the infant’s attention was placed in between every fourth item. Attention grabbers consisted of a starry sky with stars moving randomly and birds that popped out and chirped (total duration about 3 s).

A total of 50% gaze data in each trial (from -500 ms until the end of the analysis window) was required for inclusion. In Study 1 infants on average contributed 5.75 trials (from a maximum of 8) in the spider condition, 5.63 trials in the flower condition, 5.31 trials in the snake condition, and 5.38 trials in the fish condition. Participants with 1–3 valid trials in one condition were still included because mixed models control for an uneven distribution of trials.

#### Data Analysis

**FIGURE 3:**
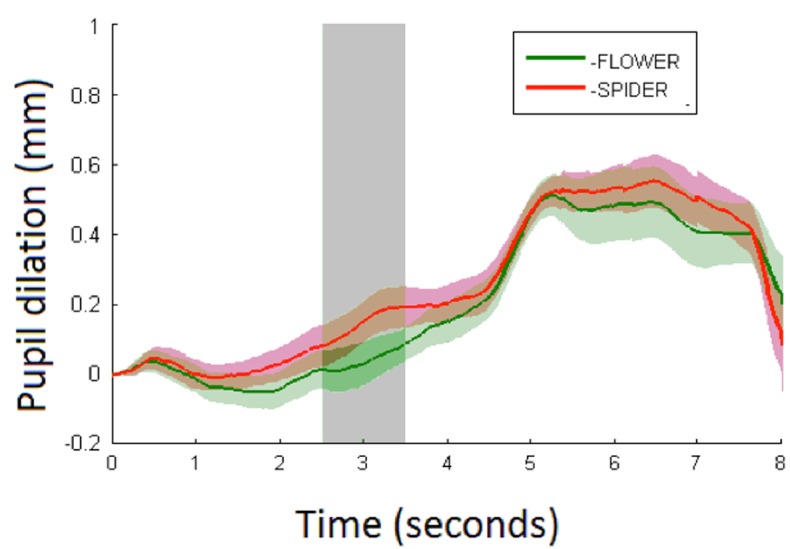
Average pupil dilation (mm) for spiders (red) and flowers (green) over 8 s with shaded areas showing the standard errors. Stimulus onset is at 0 s with a duration of 5 s at which a white screen onsets. The analysis time period is marked in light gray.

Data was imported into TimeStudio^1^ (Nyström et al., 2016), an open source analysis environment for eye tracking data and general time series analysis accessible from MATLAB. Spurious data samples were removed based on the second derivate and minor gaps in the data were interpolated (max 5 samples at 60 Hz). A moving average filter (width 5 samples at 60 Hz) was applied and data was baseline corrected based on the average pupil size for the first 100 ms of the stimulus presentation. The analysis interval ranged from 2.5 to 3.5 s after stimulus onset; during this time the mean pupil dilation was assessed. The selected time-window is consistent with pupil dilation effects found in previous infant studies occurring or starting around 2 s after stimulus onset (e.g., Geangu et al., 2011; Gredebäck et al., 2012; Sirois and Jackson, 2012). The analysis script including all data (from Study 1), settings and scripts can be downloaded using uwid ts-429-125 inside the TimeStudio environment. Running this uwid will recreate **Figure 3**.

Statistical analysis was performed using a linear mixed model in R version 3.0.2 (Ihaka and Gentleman, 1996) using lmer in the lme4 package (Bates et al., 2015). In all analyses we used pupil dilation as dependent variable, trial number as a continuous predictor (1–8), experiment as a categorical predictor (spider vs. flower, respectively, snake vs. fish), and participant as random factor. The *p*-values of the fixed factors are based on Satterthwaite approximation of degrees of freedom. The spider-flower experiment and the snake-fish experiment in Study 1 were conducted within-participants, but will be reported with separate analyses due to luminance differences between the category-pairs preventing a direct statistical comparison.

### Results

#### Spiders vs. Flowers

Pupillary responses are depicted in **Figure 3**. The linear mixed model (**Table 1**) demonstrates a main effect of condition (*p* < 0.01) with larger pupil dilations for spiders (mean = 0.14 mm, CI_95_ = 0.08–0.19) than flowers (mean = 0.03 mm, CI_95_ = -0.03–0.09). No effect of trial or interaction between condition and trial was observed. Results speak to increased arousal in response to spiders compared with flowers in 6-month-old infants.

**Table 1:** Fixed effects of condition, trial, and condition × trial on pupil dilation.

	β	Std. Error	*t*-value	*p*
Intercept	-0.22	0.11	1.93	0.06
Condition	0.18	0.07	2.63	<0.01^∗∗^
Trial	0.05	0.03	1.68	0.10
Condition × trial	-0.02	0.02	-1.40	0.16

^∗^*p < 0.05, ^∗∗^*p* < 0.01*.

**FIGURE 4:**
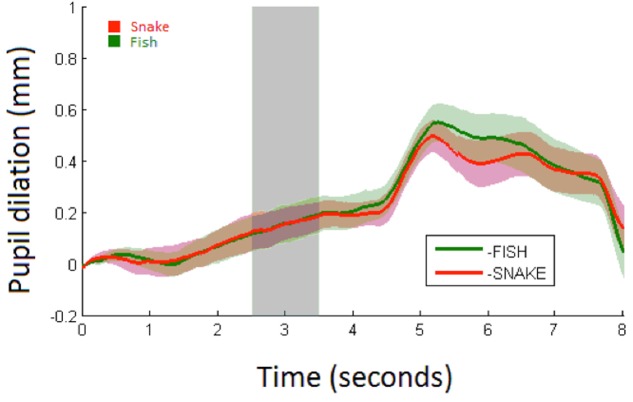
Average Pupil dilation (mm) for snakes (red) and fish (green) over 8 s with shaded areas showing the standard errors. Stimulus onset is at 0 s with a duration of 5 s at which a white screen onsets. The analysis time period is marked in light gray.

Snake vs. Fish. Pupillary responses are depicted in **Figure 4**. The linear mixed model (**Table 2**) did not show a significant main effect of condition (*p* = 0.53; mean pupil dilation for snake = 0.16, CI_95_ = 0.04–0.27, and fish = 0.16, CI_95_ = 0.06–0.25), trial, or an interaction between condition and trial.

**Table 2:** Fixed effects of condition, trial, and condition × trial on pupil dilation.

	β	Std. Error	*t*-value	*p*
Intercept	0.08	0.12	0.65	0.52
Condition	0.15	0.08	0.63	0.53
Trial	0.03	0.03	0.87	0.39
Condition x trial	-0.02	0.02	-0.99	0.32

### Discussion

Results of the spiders-flowers experiment were in line with our hypothesis of increased pupillary dilation for ancestral threats compared to non-threatening stimuli in infants. As pupillary dilation reflects arousal, this early physiological response may contribute to the increased probability for forming fear-associations with spiders compared to neutral stimuli such as flowers and mushrooms reported in human adults (Öhman and Mineka, 2001; Mineka and Öhman, 2002) and infants (Rakison, 2009; Hoehl and Pauen, 2017).

However, results of the snake-fish experiment suggest that there was minimal difference in infants’ physiological arousal in response to these stimulus categories. Although a direct comparison between category pairs is not possible as we matched luminance across stimuli only within pairs, it is notable that infants’ pupillary responses for snakes and fish were very similar to their responses to spiders and thus higher than their responses to flowers in the spider-flower experiment. This could indicate either that infants react with increased arousal to animate stimuli in general (hinting at a possible “life detector” mechanism) or that their responses to snakes generalized to the perceptually matched fish in this within-participant study. In Study 2 we therefore measure infants’ responses to snakes and fish using a between-participant design in order to rule out potential carry-over effects.

## Study 2

In Study 2 pupillometric data from one group of participants viewing only snakes were compared with pupillometric data from another group viewing only fish.

### Materials and Methods

#### Participants

Study 2 included 32 6-month-old infants (*M* age = 184 days, *SD* = 2.82, 9 boys), i.e., 16 infants per experiment. All participants were included in the final analysis. As in Study 1, participants were recruited by means of their parents responding to an invitation letter sent to families with children of appropriate age.

#### Material and Apparatus

Stimuli from the snake-fish experiment from Study 1 were used.

#### Procedure

Procedures were the same as in Study 1, with the exception that each infant only saw pictures from one stimulus category. Infants on average contributed 5.56 trials in the snake condition and 5.31 trials in the fish condition.

### Results

#### Snake vs. Fish

**FIGURE 5:**
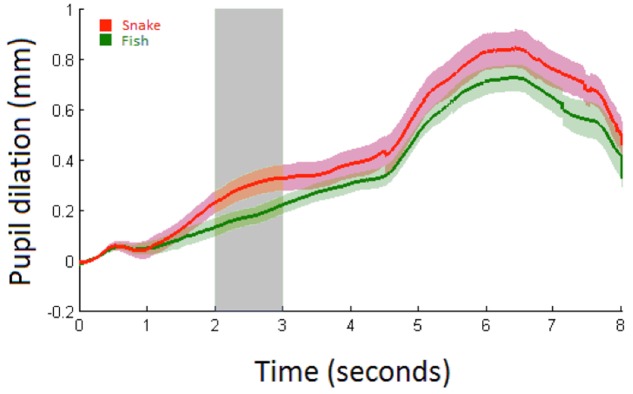
Average pupil dilation (mm) for snakes (red) and fish (green) in Study 2 over 8 s with shaded areas showing the standard errors. Stimulus onset is at 0 s with a duration of 5 s at which a white screen onsets. The analysis time period is marked in light gray.

Pupillary responses are depicted in **Figure 5**. The linear mixed model (**Table 3**) demonstrated a significant main effect of condition (*p* = 0.04; mean pupil dilation for snake = 0.29, CI_95_ = 0.25–0.33, and fish = 0.17, CI_95_ = 0.14–0.20). No significant effects were observed for trial or interaction between condition and trial.

**Table 3:** Fixed effects of condition, trial, and condition × trial on pupil dilation.

	β	Std. Error	*t*-value	*p*
Intercept	0.06	0.09	0.64	0.52
Condition	0.12	0.06	1.99	0.04^∗^
Trial	>-0.01	<0.01	-0.07	0.95
Condition × trial	>-0.01	<0.01	-0.02	0.99

^∗^*p < 0.05, ^∗∗^*p* < 0.01*.

## General Discussion

We measured 6-month-old infants’ pupillary responses when viewing stimuli representing ancestral threats (spiders and snakes) and visually matched neutral control stimuli (flowers and fish). Infants responded with increased pupillary dilation to pictures of spiders and snakes when compared with pictures of flowers and fish. Sympathetic pupillary dilation is directly linked to activity in the noradrenergic system (Gilzenrat et al., 2010), arousal and increased focused attention (Laeng et al., 2012). In summary, our results support the notion of an evolved mechanism that is sensitive to spiders and snakes. Six month old infants react with increased physiological arousal to these ancestral threats compared to non-threatening control stimuli.

In Study 1, we found larger pupil dilation for spiders than for luminance- and color-matched flowers in 6-month-olds. Though in line with a previous study showing that infants look longer toward spiders than non-threatening control-stimuli (Rakison and Derringer, 2008), this finding is novel in that it provides first evidence that infants respond to the sight of a spider with increased arousal. We contrasted spiders with flowers in order to keep in line with previous research (Rakison and Derringer, 2008; Rakison, 2009). However, this means that infants’ arousal to spiders might also be attributed to the detection of an animate being, thus possibly hinting at a mechanism responsive to animals in general. We therefore contrasted snakes with fish in another experiment.

In the within-participants snake-fish experiment in Study 1 we observed no differences in infants’ pupillary dilation to both categories. Responses to snakes and fish were similar to infants’ responses to spiders. In line with a potential “life detector” mechanism, one possible explanation is that our participants did not differentiate between snakes and fish and that animals in general elicit arousal in infants. Alternatively, infants in the snake-fish experiment may have been aroused specifically by the snake stimuli but this response carried over to the visually closely matched fish which were shown intermediately in randomized sequence. This, of course, would imply that some form of generalization can take place from ancestral threats to perceptually similar non-threatening stimuli. In order to distinguish between both interpretations we carried out Study 2 with fish and snakes shown to separate groups of infants in a between-participants design to avoid potential carry-over effects.

In Study 2 infants in the snake condition reacted with significantly increased pupillary dilation compared to infants in the fish condition speaking to a specific sensitivity to snakes as an ancestral threat in 6-month-old infants. Although luminance differences across category pairs impede direct statistical comparisons, it is notable that across experiments infants showed the smallest pupillary response to flowers (0.03 mm) followed by spiders (0.14 mm) and fish (and snakes in Study 1:0.16 mm) with a particularly increased response to snakes presented in isolation (0.29 mm in Study 2). Thus, infants seem to be aroused by images of animals, but ancestral threats, especially snakes, elicit a particularly strong reaction. To what extent a life-detector mechanism potentially explains pupil dilation remains a question for future research. However, Study 2 also specifically shows that pupil dilation is greater for snakes than fish

Our results extend earlier findings of quicker visual detection of snakes compared to flowers in 8- to 14-month-old infants (LoBue and DeLoache, 2010). Not only do infants allocate visual attention more quickly to snakes than other stimuli, they also react to snakes and spiders with physiological arousal indicating involvement of the noradrenergic system. This result is also in line with the finding of an enhanced startle eye blink response when infants listen to evolutionary fear-relevant sounds including snake hissing compared to modern threats or pleasant sounds (Erlich et al., 2013).

Taken together, findings from this and several other studies (Rakison, 2009; LoBue and DeLoache, 2010; Erlich et al., 2013; Hoehl and Pauen, 2017) provide growing cumulative evidence for an evolved mechanism that ensures special attention and facilitated fear-learning for ancestral threats in early human ontogeny (Seligman, 1971). It is notable, though, that there is little evidence for 18- to 36-month-old toddlers displaying fear to or spontaneously avoiding live snakes and spiders (Lobue et al., 2013). Thus, there is currently limited evidence for an evolved full-fledged fear response as suggested by Poulton and Menzies (2002), unless one assumes that by 18 months most infants have already habituated to snakes and spiders. Rather, most researchers seem to agree that early attention biases and arousal to ancestral threats predispose humans to develop specific fears of these stimuli given appropriate direct or vicarious learning opportunities (Rachman, 1977) in the sense of an evolved probabilistic cognitive mechanism (Bjorklund and Ellis, 2014; Bjorklund, 2015).

Some limitations of the current study should be noted. First, although the number of stimulus exemplars used in the current study (8 per category) is consistent with or even higher than in previous behavioral research on the same topic (Rakison, 2009), categories should be represented more comprehensively in future studies. Though stimuli were matched in a range of relevant low-level features, the spider and flower stimuli in particular were not perfectly matched in terms of features and complexity, as the use of ecological stimuli was of great importance to us in the current study. Future research may address this issue by using a set of stimuli based on schematic illustrations of spider-like vs. not spider-like arrangements of a “body” and “legs” or “petals” (e.g., New and German, 2015). Furthermore, due to the relative novelty of using pupillary dilation in infancy research, it is difficult to interpret some of the characteristics of the response such as its latency. It is conceivable that the timing of pupillary reactions will prove informative on infants’ cognitive processes in the future, but it is necessary to acquire more data in different paradigms in order to be able to draw firm conclusions on this. Finally, although we deem 6-month-olds unlikely to have been exposed to spiders and/or snakes or experienced direct fear-conditioning or social learning of specific fears, we cannot know for sure that infants included in our study were unaffected in their responses by prior experience.

## Conclusion

We provide evidence that infants at 6 months of age respond with increased arousal, as indicated by pupillary dilation, to spiders and snakes compared with flowers and fish. We suggest that stimuli representing an ancestral threat to humans induce a stress response in young infants. These results speak to the existence of an evolved mechanism that prepares humans to acquire specific fears of ancestral threats.

## Author Contributions

SH and GG conceived of the experiments. KH and MJ performed the experiments and data analyses. All authors contributed to the interpretation of the results. SH wrote an initial version of the manuscript; all authors provided comments and approved of the final version.

## Conflict of Interest Statement

The authors declare that the research was conducted in the absence of any commercial or financial relationships that could be construed as a potential conflict of interest.

## Footnotes

1 www.timestudioproject.com
